# Is Early Puberty Triggered by Catch-Up Growth Following Undernutrition?

**DOI:** 10.3390/ijerph9051791

**Published:** 2012-05-09

**Authors:** Lemm Proos, Jan Gustafsson

**Affiliations:** Department of Women’s and Children’s Health, Uppsala University, SE-751 85 Uppsala, Sweden; Email: jan.gustafsson@kbh.uu.se

**Keywords:** undernutrition, stunting, nutritional rehabilitation, adopted children, catch-up growth, early puberty

## Abstract

Undernutrition during fetal and postnatal life is still a major problem in many low- and middle-income countries. Even in high-income countries malnutrition may exist in cases of intrauterine growth retardation, as well as in chronic conditions such as anorexia nervosa and inflammatory bowel disease. Children adopted from developing countries are often chronically malnourished. Nutritional rehabilitation, resulting in catch-up growth, is often complicated by influences originating in fetal life as well as during postnatal growth. This may result in hormonal and metabolic changes as well as alterations in pubertal development. The present review focuses on fetal, postnatal and fetal-postnatal undernutrition and subsequent catch-up growth as well as catch-up growth in relation to pubertal development. Catch-up growth in children can be associated with early puberty following fetal or combined fetal-postnatal undernutrition. However, early puberty does not seem to occur following catch-up growth after isolated postnatal undernutrition. Gonadotropins have been reported to be elevated in prepubertal adopted girls as well as during catch-up growth in animals. Even if other factors may contribute, linear catch-up growth seems to be associated with the timing of pubertal development. The mechanisms behind this are still unknown. Future research may elucidate how to carry out nutritional rehabilitation without risk for early pubertal development.

## 1. Introduction

Undernutrition, caused by lack of food and/or the wrong composition of food, is a world-wide problem, causing impaired growth and pubertal development primarily in the developing world, but also, to a lesser extent, in affluent countries. In developing countries undernutrition is often combined with other conditions such as infections or malabsorption. Since it has been shown that well-nourished populations all over the world grow in a fairly similar manner, the WHO has established reference populations [1,2] in relation to which nutritional status can be defined in a population or an individual [2,3]. According to the definitions accepted by WHO, linear growth below –2 SD in height for age denotes chronic undernutrition resulting in stunting. Weight for height below –2 SD indicates wasting, which is a sign of acute undernutrition. There are also reference values for BMI for age for the preschool years [1] and from birth to 18 years [4] which combine height and weight parameters.

The aim of nutritional rehabilitation is improved child growth following stunting which results in a period of accelerated growth, wholly or partly compensating for the previous slower growth rate. The accelerated growth is called catch-up growth [5,6,7,8,9,10,11]. Linear catch-up growth may be preceded by a period of catch-up weight gain [7]. Unless specified otherwise, the term “catch-up growth” refers to linear growth in this paper.

Undernutrition is still a major problem in many low- and middle-income countries. In India, where the economic development is accelerating, 48% of the children below five years of age are still stunted due to chronic undernutrition [12,13]. This contributes heavily to child morbitity and is a major reason for death during childhood in developing countries. Respiratory infection and diarrheal disease, leading causes of morbidity and mortality, are commonly linked to impaired child nutrition [14,15]. In the developing world undernutrition often starts during fetal life due to maternal malnutrition, resulting in infants with low birth weight. Their postnatal life is frequently associated with growth retardation as well as delayed pubertal development.

In high-income countries, in spite of access to adequate nutrition, undernutrition still poses a problem particularly in the form of intrauterine growth retardation (IUGR) which results in infants being born small for gestational age (SGA). The work of Barker *et al.* [16,17,18] and other groups [19,20] have shown that growth impairment during fetal life may increase the risk of cardiovascular disease, hypertension and deranged glucose metabolism later in life.

In affluent societies undernutrition can also appear postnatally, due to inflammatory bowel disease, anorexia nervosa, asthma or other chronic diseases. 

In many developing countries, as in India, living conditions are rapidly improving, and previously chronically undernourished children now have access to better nutrition [14,21,22]. However, nutritional rehabilitation may be associated with complications. Thus, children living under conditions representing a transition from low-income to high-income situations, may be at risk of developing obesity and type 2 diabetes [21,23]. Several questions remain to be answered regarding how nutritional rehabilitation should best be carried out [14]. 

Another transitional situation occurs when children are adopted from underprivileged circumstances where they have become chronically undernourished to a country where they have good access to food and health care. Such a favourable situation for nutritional rehabilitation may not be uncomplicated, however. Studies on children adopted from developing to high-income countries have demonstrated that catch-up growth after adoption can be associated with early pubertal development. Precocious puberty increases the risk for short adult height due to premature closure of the epiphyseal growth plates. In addition, early puberty may also lead to psychosocial difficulties. The mechanism behind the development of early/precocious puberty following catch-up growth is still unknown.

Undernutrition may occur during different time periods in life, and nutritional rehabilitation is not simply a matter of compensating for the lack of proteins, carbohydrates, lipids and micronutrients. Increased understanding of the actual state of undernutrition, and what happens metabolically during nutritional rehabilitation is essential for the design of relevant treatment as well as preventive care of mothers and children. With this in mind we will review some human and animal studies describing various types of undernutrition, subsequent catch-up growth and associated hormonal changes. A special focus will be on early pubertal development. It seems helpful to categorize undernutrition as being fetal, postnatal or combined fetal-postnatal and to discuss growth and pubertal development in each of these conditions. 

Thus, the aim of this article is:

(1) To review research on catch-up growth after chronic undernutrition.
(2) To review the relationship between catch-up growth and early pubertal development.

## 2. Fetal Undernutrition in Relation to Later Growth and Puberty

Undernutrition can already start during fetal life. In the intrauterine environment, the growth of the fetus can be retarded due to a number of causes [24]:

- Lack of nutrients due to deficient maternal nutrition. This is the major cause in the developing world.
- Placental defects.
- Fetal disease
- Maternal disease

Intrauterine growth restriction (IUGR) can be detected by repeated ultrasound measurements to record fetal growth. In IUGR there is a reduction of the expected fetal growth rate. Approximately 4–8% of newborn infants in high-income countries and up to 30% in developing countries are identified as growth restricted [25]. Being small for gestational age (SGA) is defined as having a birth weight < –2 SD [26]. An SGA birth may be the result of chronic undernutrition *in utero*. 

### 2.1. Catch-Up Growth

Low birth weight infants born in developing countries, most often will continue postnatal life in a resource-poor environment with deficient nutrition and frequent exposure to infectious disease. Under such conditions catch-up growth may not occur.

If, on the other hand, such infants are born in a developed country where living conditions are good, it is possible to study the effect of nutritional rehabilitation following fetal undernutrition:

In a study from Sweden [27] it was shown that of children born SGA (birth weight < –2 SD) 87% had catch-up growth reaching above –2 SD within 2 years, and attained normal or early puberty. The remaining 13% did not catch up and remained below –2 SD throughout childhood, entering puberty at a normal age or somewhat early.

In another Swedish study [28] of children born SGA (66 boys and 57 girls) followed up from birth to four years of age it was shown that the boys and two thirds of the girls had fast weight catch-up during the first three months. Length catch-up, however, was more gradual and continued over the follow-up period. At the end of the four year period only six boys and three girls remained below –2 SD for both weight and height.

### 2.2. Pubertal Development

Several reports indicate a strong association between prenatal growth and the rate of pubertal development, at least for girls [29,30,31,32,33,34]. In order to examine the hypothesis that perinatal factors influence the onset of puberty Persson *et al.* [29], in a population-based study, compared 550 children “exposed” to various perinatal conditions with 688 normal children. The children were all born as singletons in Uppsala, Sweden in 1973–1977. The data showed that girls born SGA were 5 months earlier in their pubertal development than those born appropriate for gestational age (AGA). There was no corresponding difference for boys. This study is one of the first to establish the association between being born SGA and early pubertal development in girls. The careful selection criteria and the fairly large population base represent strengths of this study.

 Similarly, Lazar *et al.* [30] reported that age at onset of puberty was significantly lower in persistently short SGA children as compared to children born AGA. Moreover, menarche occurred significantly earlier in the SGA girls compared with those born AGA. 

Several studies on humans have indicated that impaired fetal growth may have a long-lasting effect on pubertal development. Thus, Ibanez and de Zegher [31,32] and Ibanez *et al.* [33,34] reported that there is increasing evidence for a link between prenatal growth and pubertal development. According to the research reviewed by the authors, menarche occurs early in girls who had an early/normal pubertal onset and who experienced prenatal growth restriction followed by rapid catch-up growth with hyperinsulinemia and excess adiposity. Moreover, low birth weight may increase the risk for precocious pubarche, and polycystic ovary syndrome, including ovarian hyperandrogenism, dyslipidemia, and hyperinsulinism [35]. 

Weight gain during infancy and childhood influences the timing of pubertal development [36,37]. An early adiposity rebound may be associated both with later obesity and early menarche [38,39]. The respective roles of development of obesity *vs*. that of increased linear growth for pubertal maturation remain to be clarified [37,39]. 

Engelbregt *et al*. [40] investigated whether intrauterine malnutrition or immediate postnatal food restriction affect the onset of puberty in male and female rats. They found that malnutrition during late gestation or directly postnatally resulted in delayed onset of puberty in IUGR and food restricted male rats as well as in IUGR female rats (but not in food restricted female rats). The onset of puberty in the growth retarded rats and in their controls did not depend on the achievement of a certain crucial weight.

Léonhardt *et al*. [41] have shown that in rat pups, the onset of puberty is delayed in both sexes when the mother is exposed to food restriction during both gestation and lactation as well as during lactation alone. 

Woelfle [42], concludes in a review that a number of studies indicate a close association of weight catch-up of SGA-children and early pubertal development.

Recently, Wehkalampi *et al.* [43] reported a follow-up study of children born preterm. The study included 188 children with very low birth weight (VLBW) and 190 controls born at term. They analysed the growth data retrospectively and found that the pubertal growth spurt occurred significantly earlier in preterm VLBW subjects, regardless of whether they were born SGA or AGA. The authors speculate that early pubertal development may influence adult metabolic outcomes in children born VLBW. 

Thus:

- *Infants born into a resource-poor environment may not undergo catch-up growth due to nutrient deficiency and diseases, particularly infections.*
- *Postnatal catch-up growth is possible under favourable conditions for the majority of undernourished infants born SGA.*
- *Prenatal growth restriction followed by postnatal catch-up growth may be associated with earlier timing of puberty.*
- *Girls born SGA and children born preterm with VLBW are prone to early pubertal development.*

## 3. Postnatal Undernutrition, Growth and Puberty

Children, born AGA, may suffer postnatal undernutrition as a result of disease or an inadequate diet. Retarded growth and delayed onset of puberty are common conditions in children with chronic disease [44].

### 3.1. Gastrointestinal Disease

#### 3.1.1. Crohn’s Disease

Growth impairment and pubertal delay are frequent complications of inflammatory bowel disease, particularly Crohn’s disease. Chronic malnutrition is caused mainly by inadequate food intake and proinflammatory cytokines. Various treatments are available: corticosteroids, optimization of immunomodulatory drugs, enteral nutrition and, if necessary, surgery for ulcerative colitis and for intestinal complications of localized Crohn’s disease. Biologic agents can further be used for mucosal healing. Following adequate therapy catch-up growth can take place. It is important to utilize the “window of opportunity” for growth before puberty advances too far [45]. 

#### 3.1.2. Celiac Disease

Patients with celiac disease may present with short stature [46]. When treated with a gluten-free diet, these children show an accelerated growth rate, with peak growth velocity occurring during the first year. The catch-up growth may not be completed after three years. Bosio *et al*. [46] reported that puberty started at a normal age.

### 3.2. Asthma

Growth problems sometimes occur in children with poorly controlled asthma [47]. There may be a chronic delay of both growth and puberty. Systemic corticosteroids suppress growth for as long as the treatment is maintained. When treatment is withdrawn, catch-up growth may occur, and partly compensate for the growth retardation. Inhaled corticosteroids may also suppress growth, depending on the dose and administration regimen.

### 3.3. Eating Disorders

Swenne [48] has demonstrated that girls afflicted with eating disorders are growth retarded. This may be followed by catch-up growth after treatment. Since this may take many years, some loss of adult height is often a consequence of the eating disorder. Consistent with this Lanzouni *et al.* [49] reported that patients with anorexia nervosa and stunting undergo catch-up growth, although not complete, after treatment. Similar results have been reported by Modan-Moses *et al.* [50].

Following treatment, girls with eating disorders and primary amenorrhea progress rather slowly through puberty [51]. The weight required to reach menarche can be predicted by the prepubertal weight which may represent the individual’s normal growth track unaffected by the eating disorder.

IGF-1 has been identified as a useful indicator for monitoring the nutritional rehabilitation [52].

### 3.4. Early Postnatal Undernutrition and Critical Periods

If rats are fed a protein-energy deficient diet, total pancreatic beta cell weight and individual beta cell size are diminished. Thus, protein-energy malnutrition early in life may result in a diminished reserve for insulin production, which in turn may predispose to glucose intolerance or even manifest diabetes during conditions of increased insulin demand [53,54]. This may also influence the growth process.

Winick and Noble [55] have shown that in the early postnatal life in the rat there is a cell proliferation phase which lasts until weaning. During that time catch-up growth is possible. After this the number of cells does not increase, instead the cells increase in volume. It was observed that undernutrition during the cell proliferation phase could limit the number of new cells formed. Full catch-up following undernutrition during this period was not possible due to the lesser number of cells. If undernutrition took place after the final formation of all cells, only the cell content was affected and full catch-up was possible after refeeding. Thus, it follows that there may be critical periods for the potential for catch-up growth, depending on whether cellular proliferation or cellular hypertrophy predominates. 

Human fetal growth is initially due to multiplication of cells and to some degree to an increase of cell size. During the late pregnancy fetal growth mainly takes place through increase of cell size [56]. There may exist critical periods with regard to the ability for catch-up growth in humans, since studies on adopted children indicate different potentials depending on their nutritional status on arrival [57].

Thus:

- *Well-nourished children may become undernourished due to inadequate diet or disease. Their growth may be retarded and puberty delayed.*
- *After successful treatment, catch-up growth can occur, but may be prolonged. There are conflicting reports regarding whether full catch-up is possible.*
- *There may be critical periods for the potential for catch-up growth.*
- *To the best of our knowledge, catch–up growth in children, following undernutrition which occurs only postnatally, has not been reported to be associated with earlier pubertal development.*

## 4. Combined Fetal-Postnatal Undernutrition, Growth and Puberty

Low birth weight (LBW) due to maternal malnutrition, is common in the developing world where around 16% of infants are estimated to have a birth weight <2500 g [58]. In South-Central Asia 27% are born with LBW [58]. Thus, a great number of children begin their life chronically undernourished. In a developing country the LBW child mostly has to continue with limited access to food and may later in childhood have to participate in energy demanding labour, necessary for the survival of the family. These children thus represent combined fetal and postnatal undernutrition. In these populations the children grow on average along –2 SD in height according to the WHO [1,2,3,58] reference. In addition, they enter puberty relatively late compared to privileged children. Their adult height corresponds to about –2 SD. However, it has been shown that the pubertal growth component is similar both in undernourished and privileged groups of boys. This means that the height gain during puberty is the same in both groups. The lower adult height of the chronically undernourished boy is thus due to a lower height at the start of puberty [59].

### 4.1. Catch-Up Growth

In a review of studies on stunted children, Martorell *et al*. [60,61] reported that these children continue to be stunted if they remain in the environment in which they became chronically undernourished. Moreover, the potential for catch-up growth increased as maturation was delayed and the growth period prolonged. In addition, the effect of a poor environment leading to continued stunting despite a period of treatment for malnutrition, has been pointed out by Graham *et al*. [62]. 

International adoptions offer possibilities to study undernourished children in an environment where food supply is abundant, which in turn leads to catch-up growth. Proos *et al.* [63,64] studied 114 boys and girls adopted in Sweden from India at arrival and during a follow-up period of 2 years. The majority of the children were stunted on arrival in Sweden and catch-up growth was observed continuously during the 2 years, with no signs of levelling off. In 37 cases the birth weight was known. Thirty (81%) of these had a birth weight <2500 g. Although data on birth weight were lacking for the remaining children, it is likely that the majority also had a low birth weight and thus were subject to combined fetal-postnatal undernutrition. 

Golden [10] has reviewed studies regarding whether complete catch-up is possible after malnutrition, and has reported that catch-up growth appears to be adequate under favourable circumstances. However, it can be interrupted by an early pubertal development.

### 4.2. Pubertal Development

Kulin *et al*. [65] compared 342 privileged urban children in Nairobi, Kenya with 347 impoverished rural adolescents. They found that the poor children had a delayed pubertal maturation. The authors also showed [66] that the levels of gonadotropins in urine from rural adolescents were lower than from those living in urban areas. However, following matching for pubertal stages there were no significant differences in the levels of gonadotropins.

Gupta and Singh [67] have reported that girls from rural areas were shorter and weighed less as compared to those from urban slums. At a certain age a higher degree of pubertal development was associated with a higher mean body weight and height. Quamra *et al.* [68] reported that girls with an insufficient energy intake had approximately one year later onset of breast and pubic hair development. The authors concluded that onset of puberty, although under genetic control, is strongly influenced by the environment. 

Adair [69] studied 997 girls from rural and urban communities in the Philippines. Girls who were long and thin at birth reached menarche up to 6 months earlier than did girls who were short and light. Thinness at birth had the most pronounced effect on menarche among girls who grew rapidly during the first 6 months of life. The data confirm that programming of postnatal growth and maturation is established already in utero. 

### 4.3. Adoption

Adolfsson and Westphal [70] reported in 1981 on observing early pubertal development in seven girls adopted from Far-Eastern countries.

In order to investigate the occurrence of early puberty in adopted children Proos *et al.* [71,72] studied 107 girls from India adopted in Sweden through the same adoption agency and born 1971 or earlier. The study was the first to analyse the characteristics of a large population of adopted girls, selected only according to time of birth and country of origin, enabling comparison with national growth references.

The majority was stunted on arrival and subsequently underwent catch-up growth. This continued until the start of the pubertal growth spurt, which occurred about 1.5 years earlier than that of the reference population. The girls, as a group, had menarche 1.2–1.5 years earlier than well-nourished reference populations in India and Sweden [72]. Analysis of the pubertal growth component showed that it was normal with regard to extent and duration, but also that it started 1.5 years earlier than that of the reference population [73,74]. In addition, the girls with the most pronounced stunting and fastest catch-up growth had the lowest menarcheal age [75]. Interestingly, menarche occurred at similar height and weight in the adopted girls as in girls in an Indian underprivileged reference population, even though the start of puberty was delayed almost three years in the latter group [71]. Further, the two groups ended up with similar adult heights. The common denominator for the two populations was the initial chronic undernutrition.

Several other researchers have also reported occurrence of early or precocious puberty among internationally adopted children [76,77,78,79,80,81,82,83,84,85]. Virdis *et al.* [77] studied 19 adopted girls, who were examined due to early puberty. The girls who were above five years at arrival had the earliest development. The authors hypothesize that catch-up growth leads to development of early puberty and propose that fat accumulation contributes by increasing availability of estradiol.

In a large population-based study in Denmark, Teilmann *et al.* [86] reported that internationally adopted girls run a 10–20 times increased risk of developing precocious puberty. They also showed that 5–8 years old adopted girls had increased levels of gonadotropins without any clinical signs of puberty. The authors concluded that the early onset of puberty in adopted girls is of central origin [87]. The studies are important since they define the risk for precocious puberty in internationally adopted girls and add to the knowledge of the hormonal status during their prepubertal period. 

Thus:

- *Children who have been undernourished in utero and continue life in an environment where food is scarce, will not experience catch-up growth. They will enter puberty later than a well-nourished reference population.*
- *On the other hand, chronically undernourished children who are transferred to an environment with abundant food supply will undergo catch-up growth of varying degrees, and may develop early puberty.*
- *The timing for the onset of puberty is dependent on the degree of undernutrition as well as the rate of catch up growth.*
- *Prepubertal adopted children have been reported to have elevated gonadotropins.*

## 5. General Discussion

This review focuses on different aspects associated with triggering of early puberty following undernutrition and subsequent catch-up growth. In our opinion the major areas of relevance for the understanding of the causation of early pubertal onset are those discussed below.

### 5.1. Metabolic Signalling, Nutritional Supply and the Onset of Puberty

Studies on metabolic signalling indicate links between supply of nutrients and the development of reproductive function. Even though leptin does not initiate puberty, it has an important permissive role in pubertal development. Ghrelin is an orexigenic signalling compound, which also influences GH secretion. There is evidence that ghrelin may take part in the regulation of gonadotropin secretion and thus influence the timing of puberty. PYY3-36 is another gastrointestinal hormone proposed to be involved in the control of food intake and energy balance. In addition PYY3-36 modulates GnRH and gonadotropin release. Moreover, the hypothalamic KiSS-1 system is sensitive to nutritional status, and may influence reproductive function during negative energy balance [88,89,90,91].

Martos-Moreno *et al.* [92] have reviewed the effects of over- and undernutrition on metabolic signalling in human puberty. The roles of ghrelin, leptin and other adipokines are discussed. The results link the energy status of the body to its sexual development. The authors particularly point out that peptides, produced in the digestive tract and adipose tissue provide the central nervous system with information regarding metabolic status. These peptides play an important role in modulating the gonadotropic axis. Their absence or an imbalance in their secretion may disturb the onset or progression of puberty.

### 5.2. Endocrine Disruptors and Early Pubertal Development

Endocrine-disrupting chemicals have been suggested to be an environmental cause of early pubertal development in several populations including adopted girls [93,94,95]. In a study from Belgium [96] high levels of p,p'-DDE were demonstrated in 26 immigrant girls with precocious puberty as compared with only two of 15 native Belgian girls. One hypothesis is that exposure to weakly estrogenic DDT, in the countries of origin, could stimulate hypothalamic maturation in parallel with a feedback inhibition of pituitary gonadotropins. The inhibition of gonadotropins would in turn prevent the manifestation of central maturation. After migration to the country of adoption withdrawal from exposure would cause disappearance of the negative feedback, allowing onset of puberty. This hypothesis is supported by experimental work on the effect of DDT in rodents [97].

Contaminating toxic substances in the environment evidently can disturb the pubertal developmental process [93,94]. With increasing industrialization and use of pesticides world-wide, sources of contamination multiply, creating risks for dangerous exposition for expectant mothers and young children in developing as well as in high-income countries, necessitating increased vigilance against these dangers [98]. Still, although endocrine disruptors may cause some of the pubertal developmental aberrations, these compounds may not be the whole explanation. Soriano-Guillén *et al.* [84] and Teilmann *et al*. [86] did not report significantly increased risk for precocious development in children who had immigrated with their families. We also have to consider animal experiments including refeeding after food restriction, which show accelerated sexual maturation associated with catch-up growth [99,100]. It seems unlikely that the maturation of laboratory animals with controlled food intake has been disturbed by toxic substances. 

### 5.3. Catch-Up Growth and Pubertal Development

As described above, catch-up growth can take place following intrauterine undernutrition, after postnatal undernutrition of well-nourished children as well as after combined fetal-postnatal undernutrition.

The catch-up growth may be incomplete in cases of severe undernutrition but may also be limited by the appearance of early puberty, which can cut short the catch-up growth process [71,72,101].

Following combined fetal-postnatal undernutrition the initial status before nutritional rehabilitation strongly influences the outcome. Although catch-up growth takes place, the children with more initial stunting do not attain the same adult height as those with a better start regarding nutritional status [71,102]. 

During the Dutch Hunger Winter in 1944–1945 many expectant mothers starved. Effects on the birth weigths of their offspring have been reported varying with the trimester in which they were exposed [103,104]. Several authors hypothesize that environmental conditions early in life can cause epigenetic changes that remain through life [105,106] 

In a classic study, Greulich [107,108] investigated Japanese immigrant couples in California. The immigrants were markedly shorter than the American subjects. However, the Japanese mothers, now settled in the U.S., gave birth to boys who reached an adult height similar to that of their American peers. This indicates that if maternal nutrition is optimal, already the next generation may be able to express their genetic growth potential. If the improvement of the living conditions is more gradual, as in a country in development, a secular trend in height in both sexes over the generations can be seen [109]. 

The mechanism behind the early pubertal development is not known, but there are a number of related circumstances which should be considered:

- Early puberty may occur following catch-up growth after fetal or fetal-postnatal undernutrition, *i.e.*, chronic undernutrition starting already during intrauterine life [29,71,72]. To the best of our knowledge, early puberty has not been reported after catch-up growth following isolated postnatal undernutrition in humans.
- The timing of pubertal development may be preprogrammed during early life. In a study of girls adopted from India, menarche occurred at similar height and weight as in a reference population of underprivileged girls in India. The adopted girls had a mean age at menarche of 11.8 years, compared with 14.4 years in the underprivileged group [71,72]. This may indicate a programming not linked to chronological age but to the attainment of a degree of biological maturation parallelled by a certain height and weight.
- Several groups have reported a close connection between growth acceleration and the rate of pubertal development [69,71,72,73,77,110,111,112] In a study of girls adopted from India, Proos *et al.* [75] demonstrated that the more pronounced the degree of stunting and the faster the catch-up growth, the earlier menarche ocurred. Papadimitriou *et al*. [111,112] have suggested that growth acceleration up to 2–4 years of age, is a predictor both of early pubertal development and of obesity. The authors also point out that the catch-up growth occurring after intrauterine growth retardation and that of adopted children could possibly represent similar processes.
- Tam *et al.* [113] studied the influence of prenatal and postnatal growth on the timing of menarche, and found that it occurred earlier in girls who were long and light at birth and who had higher fat mass and circulating IGF-I during childhood. Age at menarche was best predicted by combining size at birth and BMI z-score at 8 years of age.
- In order to create a situation analogous to that of undernutrition in internationally adopted children, Bourguignon *et al*. [99] studied the effect of varying the early nutritional conditions in the rat. The authors showed that rats subjected to restricted feeding had delayed hypothalamic and testicular maturation as compared to rats having access to more food. If food-restricted rats were allowed to refeed after nutritional deprivation the growth rate increased. This was associated with accelerated testicular and hypothalamic maturation. However, the effect was only observed when refeeding occurred before weaning. The findings indicate that hypothalamic maturation is influenced by variations in nutritional status and growth rate during a critical period. In addition, there was an acceleration of maturation of the glutamate receptor-dependent secretion of GnRH, suggesting the involvement of hypothalamic glutamate receptors. This is another significant paper, indicating lines of experimental research that may help in clarifying the reasons behind early pubertal development after catch-up following undernutrition.
- The importance of dietary energy for LH-pulsatility has been also been pointed out by Loucks [114] in a study on adolescent and young adult women.
- Foster and Olster [100] have reported that gonadotropins were elevated during catch-up growth in lambs. They found that severe undernutrition prevented ovulation by impairing the system governing GnRH secretion and its production of high-frequency LH pulses. Ad libitum feeding of growth-retarded lambs resulted in rapid catch-up growth, a progressive several-fold increase in LH pulse frequency and onset of reproductive cycles. The data are important, since the results are analogous to those reported for undernourished children. Moreover, it links the occurrence of catch-up growth to the simultaneous increase of LH-pulse frequency.
- As stated above, elevated gonadotropins were found in prepubertal adopted girls in a Danish study [87].

Although one must be cautious in extrapolating from animal experiments to a human context, the animal and human studies cited above indicate that the hypothalamic-pituitary-gonadal (HPG) axis becomes activated during or following catch-up growth after undernutrition.

*Thus,* *the following sequence of events seems reasonably well documented: Nutritional rehabilitation after persistent undernutrition leads to catch-up growth, coinciding with increased gonadotropin secretion, eventually leading to early puberty.*

## 6. Conclusions

This article reviews research regarding catch-up growth after the three modes of undernutrition: during fetal life, after isolated postnatal undernutrition and after combined fetal-postnatal undernutrition.

The major conclusions are:

- Following nutritional rehabilitation catch-up growth is possible after fetal, postnatal and combined fetal-postnatal undernutrition.
- Studies on adopted children arriving in their new environment at various ages show that catch-up growth can take place at any time during childhood but may be limited by critical periods.
- Early/precocious puberty following catch-up growth after fetal or fetal-postnatal undernutrition has been reported in a number of studies.
- Early pubertal development does not seem to follow catch-up growth after isolated postnatal undernutrition.
- The timing for the onset of puberty is dependent on the degree of undernutrition as well as the rate of the catch-up growth.
- Experiments in sheep have shown that catch-up growth after undernutrition is associated with an increased frequency of LH-pulses,
- Gonadotropins have been found to be elevated in adopted girls, who are still prepubertal.

It seems reasonably well documented that nutritional rehabilitation after persistent undernutrition leads to catch–up growth, coinciding with increased gonadotropin secretion, eventually leading to early puberty.

The nature of the mechanisms behind the early/precocious pubertal onset in adopted children is not yet fully known. Further studies on metabolic and endocrine status during catch-up growth following the three modes of undernutrition are needed. Such studies may eventually clarify how nutritional rehabilitation should be carried out in order to avoid early or precocious puberty.
